# Beyond “Evidence-Based Medicine” in “Detained Patients"

**DOI:** 10.1192/bjo.2022.366

**Published:** 2022-06-20

**Authors:** Farshad Shaddel

**Affiliations:** St Andrew's Healthcare, Northampton, United Kingdom. University of Oxford, Oxford, United Kingdom

## Abstract

**Aims:**

Treatments without robust evidence are not recommended. However, some patients detained in secure hospitals might need novel approaches such as: off-license use of medication, use of psychological (rather than their biological) effects of drugs. In addition, some detained patients may request for unconventional treatments they believe in. In community and capacitous patients, the clinician's role is advisory and the burden is on the patient to make the final decision and access such treatments privately. However, in a detained patient (with or without capacity), it may fall on the Responsible Clinician (RC) to deny or facilitate access to such interventions. Currently, there is no guidance for such circumstances. We have presented three real cases followed by proposing a flowchart to guide RCs.

**Methods:**

Case 1 (2019–2020): X with mild Learning Disability (LD) and mixed personality disorder detained under Section 3 with no leave to community. X asked for Hypericum which has been helpful with her headaches in the past. X had capacity to make that decision.

Case 2 (1996–97): Y with mild LD and aggressive behaviours responding instantly to any injection. Y lacked capacity so injections of distilled water was tried in his best interest, with equal positive effect. The question was about using distilled water as rapid tranquilisation with no side effects.

Case 3 (2020–21): Z with a treatment-resistant psychosis who has been unwell for months and detained in four different PICUs. Z's father requested N Acetyl Cysteine which had historical calming and sedative effects for Z.

**Results:**

The main issue in case 1 is the conflict between the patient's Human Rights and RC's Duty of care. Here the patient could be potentially deprived of their right to make an ‘unwise decision’ should the RC bar her access to a treatment which lacks evidence but is privately available to public. This can be construed as an infringement of Article 8 of Human Rights.

The issue in case 2 and 3 is rather different. Here the conflict is between the RC's duty of care to provide evidence-based treatments and the patient's “best interest” which seems to be an intervention without robust evidence.

**Conclusion:**

We have developed a flowchart to help RCs by navigating amongst several competing/ conflicting legal and ethical concepts such as: Patient's wish/Human rights, Patient's capacity, Bolam test, “Medical Treatment” Under Section 63, 62 or 58 of Mental Health Act 1983, Best interest, Second Opinion (SOAD) and advice from court.

